# In utero exposure to germinated brown rice and its oryzanol-rich extract attenuated high fat diet-induced insulin resistance in F1 generation of rats

**DOI:** 10.1186/s12906-017-1571-0

**Published:** 2017-01-21

**Authors:** Hadiza Altine Adamu, Mustapha Umar Imam, Der-Jiun Ooi, Norhaizan Mohd Esa, Rozita Rosli, Maznah Ismail

**Affiliations:** 1 Laboratory of Molecular Biomedicine, Institute of Bioscience, Universiti Putra Malaysia, 43400 UPM Serdang, Selangor Malaysia; 2 Department of Nutrition and Dietetics, Universiti Putra Malaysia, 43400 UPM Serdang, Selangor Malaysia; 3 UPM-MAKNA Cancer Research Laboratory, Institute of Bioscience, Universiti Putra Malaysia, 43400 UPM Serdang, Selangor Malaysia

**Keywords:** DNA methylation, Epigenetics, Germinated brown rice, Gamma oryzanol, Histone acetylation, Insulin resistance, Nutrigenomics

## Abstract

**Background:**

The development of insulin resistance is multifactorial, with maternal pre- and postnatal nutrition having significant influences. In this regard, high fat diet (HFD) feeding in pregnancy has been shown to increase risks of metabolic diseases. Thus, we investigated the effects of supplementation of HFD with germinated brown rice (GBR) and GBR-derived gamma oryzanol-rich extract (OE) on insulin resistance and its epigenetic implications in pregnant rats and their offsprings.

**Methods:**

Pregnant female Sprague dawley rats were fed with HFD alone, HFD + GBR or HFD + OE (100 or 200 mg/kg/day) throughout pregnancy and lactation. Their offsprings were weaned at 4 weeks post-delivery and were followed up until 8 weeks. Serum levels of adipokines were measured in dams and their offsprings, and global DNA methylation and histone acetylation patterns were estimated from the liver.

**Results:**

The dams and offsprings of the GBR and OE groups had lower weight gain, glycemic response, 8-Iso prostaglandin, retinol binding protein 4 and fasting insulin, and elevated adiponectin levels compared with the HFD group. Fasting leptin levels were lower only in the GBR groups. Hepatic global DNA methylation was lower in the GBR groups while hepatic H4 acetylation was lower in both GBR and OE dams. In the offsprings, DNA methylation and H4 acetylation were only lower in the OE group. However, dams and offsprings of the GBR and OE groups had higher hepatic H3 acetylation.

**Conclusions:**

GBR and OE can be used as functional ingredients for the amelioration of HFD-induced epigeneticallymediated insulin resistance.

## Background

The prevalence of metabolic syndrome, characterized by hypertension, abdominal obesity, T2D, dyslipidemia and insulin resistance, is on the rise. Its etiology is complex with both environmental and genetic contributions [1]. Diet is an important environmental factor that could influence the risk of insulin sensitivity, and in fact, maternal pre- and post-natal nutrition has been demonstrated to modulate risk of insulin resistance later in life [2]. Furthermore, the preference of diets that cause increased visceral fat deposition and elevated rates of lipolysis has been linked with significant rise in metabolic diseases such as insulin resistance [3]. Accordingly, maternal nutrition that promotes increased fat deposition has been shown to promote the risk of insulin resistance in offsprings especially when consumed during pregnancy. Similarly, from a nutritional point of view, bioactive compounds are able to change global DNA methylation, with implications on functional activation or repression of genes [4]. A few bioactives are specific to certain cereals such as gamma oryzanol in rice [5], which is a mix of ferulic acid esters with phytosterols [6]. It is reported to have various functional properties including cholesterol-lowering effects and antioxidative properties [7]. It was also reported to be effective in reducing weight [8], which is an important determinant of metabolic status. Moreover, excessive weight can lead to various metabolic perturbations with epigenetic implications on the risk of metabolic disease in offsprings [9].

Germinated brown rice (GBR) has higher amounts of bioactive compounds than brown rice, which confers superior bioactivity including antiobesity and antidiabetic effects [10]. In view of the current epidemic of diabetes in children and the contributions of nutrition on metabolic disease outcomes in offsprings, we hypothesized that the reported bioactivities of GBR and oryzanol may be beneficial in ameliorating high fat diet- (HFD-) induced metabolic perturbations. Moreover, we have demonstrated that GBR can ameliorate the risk of metabolic diseases, and that oryzanol is one of the important bioactives in GBR that contributes to its bioactivities [10]. Thus, in the present study, GBR and GBR-derived gamma oryzanol-rich extract were evaluated for their ability to attenuate metabolic disease induced in F1 offsprings of rats that consumed HFD during pregnancy.

## Methods

### Chemicals, enzyme-linked immunosorbent assays and epigentic kits

All solvents were of analytical grade from Merck. Biotechnolgy grade water (7732-18-5) and commercial oryzanol were obtained from Sigma-Aldrich. Rat adiponectin (Catalog No EZRADP-62 K) and insulin (Catalog No EZRMI-13 K) enzyme-linked immunosorbent assay (ELISA) kits were procured from Millipore Corporation. Rat retinol binding protein-4 dual ELISA kit (Catalog No AG-45A-0012YEK-K101) was obtained from Adipogen International, while 8-iso prostaglandin (Catlog No E-EL-R2488) and leptin (Catlog No E-EL-R0582) ELISA kits were purchased from Elabscience Biotechnology Co., Ltd. MethylFlash™ Methylated DNA Quantification Kit (Fluorometric) (Catalog No P-1035), Epiquick™ Total Histone Extraction Kit (Catalog No OP-0006), Epiquick™ Epiquick™ Total Histone H3 Acetylation Detection Fast Kit (Fluorometric) (Catalog No P-4031) and Epiquick™ Total Histone H4 Acetylation Detection Fast Kit (Fluorometric) (Catalog No P-4033) were obtained from EPIGENETIK.

### Germination of brown rice and extraction of oryzanol

Brown rice (MR220 variety) was obtained from PadiBeras Nasional Berhad (BERNAS) (Selangor, Malaysia), and was germinated as described previously [11]. Briefly, 500 g of brown rice was washed twice using tap water, after which sodium hypochlorite was added at a ratio of 1:2 (w/v) and left to soak for 30 min. It was then drained and rinsed with distilled water and soaked in hydrogen peroxide (H2O2) 1:2 (w/v) and incubated (Glass door low temperature (BOD) incubators SD-450) for 6 h at 37 °C. Then, H_2_O_2_ was drained and the rice was incubated once again in closed plastic container for 18 h at 37 °C and later oven-dried (Memmert) at 50 °C until moisture content of 8–11% was achieved using the oven dry method. We have previously demonstrated that the MR220 variety, which is commonly consumed in Malaysia, had polyhedral granular starch particles that measured around 2 to 8 μm for all starches. Its amylose content was generally less than 30%, which was significantly lowered along with its gelatinization and pasting properties after germination [12]. In this study, the GBR was ground with a stainless steel grinder (Waring Commercial) and used in preparing the rat pellets.

The extraction of gamma oryzanol from GBR was done according to Zamri [6]. Hexane (400 ml; 70%) was mixed with approximately ground GBR (100 g) and left to soak for 30 min. It was then centrifuged for 20 min at 34,800 g. Whattman No. 1 filter paper with the aid of a glass funnel was used to filter the supernatant and the extraction process repeated two more times for a total of 3 extractions. The filtered supernatants were pooled and dried using a rotary evaporator (Rotavapor® R-210, BUCHI). The oryzanol yields were reported in our earlier publication as 30.38–64.22 mg/g GBR [13].

### Experimental design

All animal experiments were implemented in accordance with the guidelines for the use of animals as approved by the Animal Care and Use Committee, Faculty of Medicine and Health Sciences, Universiti Putra Malaysia (approval number UPM/FPSK/PADS/BR-UUH/00360). Fifteen female old Sprague Dawley rats aged 8 weeks were raised on regular chow diet by the breeder were fed regular chow ad libitum and free water access during the acclimatization week. The animal house was well-ventilated with a 12 h light/dark cycle at the ambient temperature of 25–30 °C, throughout the experimental period. The animals were then grouped (*n* = 3) into high fat diet (HFD), regular chow (N), high fat diet with 50% GBR (GBR), high fat diet with high dose oryzanol extract (200 mg/kg body weight, OEHD) and high fat diet with low dose oryzanol extract (100 mg/kg body weight, OELD). Oryzanol at 100 and 200 mg/kg body weights were safe and produced metabolic changes in our earlier publications [10], hence our choice of these concentrations in the present study. Male Sprague Dawley rats that had been fed with regular chow were mated with the female rats. The female rats were maintained on these diets during pregnancy and lactation. Oryzanol extract (high and low dose) were administered via intra- gastric gavage. The high Fat Diet (HFD) formulation was made up of 47.7% total carbohydrate, 16.1% protein, 31.1% fat, 2.5% fiber and 5.1% mineral and vitamin and prepared using 50% normal rat chow powder, 24% Mazola oil, 20% Nespray full-cream milk powder, 6% sugar and 50 g of starch, placed in an oven at 60 °C for 24 h, cut into smaller pieces and then used to feed the rats. The caloric compositions of the pellets given to each group are shown in Table 1. Food intake was set at 30 kcal/100 g body weight/day for all the groups (Table 1). Accordingly, the weights of the rats were measured weekly, and the total amount of feed given daily (grams) was reviewed every week based on the new weights of the rats. After delivery, male offsprings were chosen for follow up on the effects of the perinatal interventions on insulin resistance markers. At 4 weeks post-delivery, the male offsprings (*n* = 6 per group) were weaned and maintained on regular chow (30 kcal/100 g body weight/day) for another 4 weeks. Energy efficiency of body weight (EE-BW) was calculated for the offsprings as weight gained in each group in g divided by energy intake in kJ [14]. Dams were sacrificed via when they were approximately 16 weeks old, and after weaning off the offsprings. Weekly weights of the offsprings were taken for the 8 weeks, after which they were sacrificed. Prior to sacrifice, the animals were fasted overnight and subsequently dissected after light anesthesia to harvest the liver. Blood (10 mL/rat) was collected via cardiac puncture before dissection and centrifuged at 3000 rpm for 10 min at 4 °C to separate the serum. The liver was removed immediately, washed with ice-cold saline, dried with filtered paper, and then stored in RCL2® Solution (ALPHELYS, France) at −80 °C.

**Table 1:** Animal groups and diets

Groups	High fat diet^ a^ (%)	Others	Dietary energy contributions ^a^ (Kcal/100 g pellet)
Normal	0	100% normal rat pellet	335
High fat diet	50	50% normal rat pellet	548
50% Germinated brown rice	50	50% germinated brown rice	554
Low dose OE	100	100 mg/kg/day OE	554
High dose OE	100	200 mg/kg/day OE	554

*OE* oryzanol extract. ^a^ Energy contributions are as reported in our earlier study (reference [13])

### Oral glucose tolerance test

In order to appraise the effects of the different treatments in the Dams and offsprings on systemic glucose homeostasis, oral glucose tolerance test (OGTT) was performed. Rats were initially fasted overnight prior to OGTT via intra-gastric gavage using a glucose solution (2 g/kg body weight). This was done after weaning in the dams and at 8 weeks post-delivery for the offsprings. Blood samples were taken from the tail vein at 0, 30, 60, 90 and 120 min using a glucometer (Accu-Chek, Roche Diagnostics).

### Serum insulin, adiponectin, leptin, RBP 4 and 8-iso prostaglandin

Serum levels of these markers were determined using the respective ELISA kits according to the manufacturers’ instructions. Absorbance were read on a micro plate reader at the recommended wavelengths and the results calculated from the respective standard curves; Insulin (y = 0.153x + 0.3572, *R* ^2^ = 0.9911), Adiponectin (y = 0.0026x + 0.1455, *R* ^2^ = 0.9985), Leptin (y = .2215x + 0.0286, *R* ^2^ = 0.9982), RBP 4 (y = 0.1041x + 0.2977, *R* ^2^ = 0.9875) and 8-iso prostaglandin (y = 0.0024x + 0.3167, *R* ^2^ = 0.9781).

### DNA isolation

DNA was extracted using the ZR-Duet™ DNA/RNA MiniPrep kit (Zymo Research) according to the manufacturer’s instructions. Then, the quality and quantity assessments of the extracted samples were done using Implem NanoPhotometer® prior to storage at −80 °C.

### Methylated DNA quantification (fluorometric)

Methylated DNA was quantified using a fluorometric kit according to the manufacturers’ instructions. Absorbance was read on a micro plate reader at the recommended wavelength and the result expressed as fold change with respect to the N group.

### Total histone extraction

Extraction of total histone was done according to the manufacturers’ instructions. Quantification of protein was calculated using BSA as standard curve (y = 2.3639x – 0.0234, *R* ^2^ = 0.9981).

### Total histone H3 and H4 acetylation

Total Histone H3 and H4 Acetylation were performed according to the manufacturers’ instructions. Absorbance were read on a micro plate reader at the recommended wavelength and the respective standard curves calculated (y = 8.0054x + 411.8, *R* ^2^ = 0.9618) and (y = 835.06x + 407.6, *R* ^2^ = 0.9978). The amount of acetyl histone H3 and H4 were then determined by the formula below:

$$ \mathrm{Amount}\ \left(\mathrm{ng}/\mathrm{mg}\ \mathrm{protein}\right)=\frac{\mathrm{RFU}\ \left(\mathrm{sample}\ \hbox{-}\ \mathrm{blank}\right)}{\mathrm{Protein}\ \left(\mathrm{u}\mathrm{g}\right)* \times \mathrm{slope}}\times 1000 $$

* Histone extract amount added into the sample well.

### Statistical analysis

Data were expressed as mean ± standard deviation, and the means were compared using ANOVA (analysis of variance) with Tukey’s multiple comparison test.

## Results

### Body weight

**Fig. 1:**
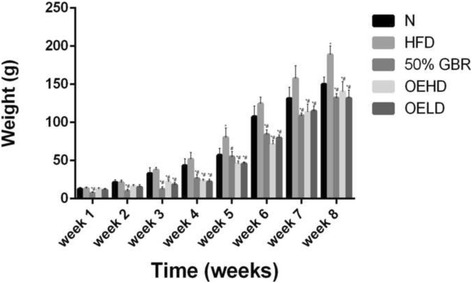
A. Average weight gains of offsprings (mean ± SD, *n* = 6) over 8 weeks. Dams were fed the respective diets for each group, while their offsprings were fed regular rat chow after weaning and observed until 8 weeks post-delivery. Abbreviations refer to the diets given to the dams: high fat diet (HFD), normal (N), high fat diet with 50% Germinated Brown Rice (GBR), high fat diet with oryzanol-rich extract (gavage) 200 mg/kg body weight (high dose) (OEHD), high fat diet with oryzanol-rich extract (gavage) 100 mg/kg body weight (low dose)(OELD). Bars representing the same week with ^*^ indicates statistical difference in comparison with HFD group (*p* < 0.05), ^#^ indicates statistical difference in comparison with N group (*p* < 0.05)

The body weights (Fig. 1) of the offsprings increased over the course of 8 weeks, with similar EE-BW for all the groups (Table 2). A significant difference (*P* =0.00001, *F* = 63.88) was observed between the HFD and treatment groups at week 8 with a percentage change of 25, 42, 34 and 43.5% in the N, GBR, OEHD and OELD respectively. Treatment groups were observed to weigh less than the N group in the first few weeks.

**Table 2:** Offsprings’ energy efficiency of body weight

Groups	Total weight gained (g)	Total food consumed (Kj)	Energy efficiency (g/Kj)
Normal	137.44 ± 7.14	698.45 ± 11.55	0.20 ± 0.06
High fat diet	175.50 ± 9.48	847.05 ± 14.10	0.21 ± 0.07
50% Germinated brown rice	124.71 ± 3.78	546.01 ± 6.95	0.23 ± 0.05
Low dose OE	127.80 ± 12.26	557.18 ± 16.70	0.23 ± 0.07
High dose OE	120.50 ± 9.90	551.24 ± 13.08	0.22 ± 0.08

*OE* oryzanol extract

### Oral glucose tolerance test

**Fig. 2:**
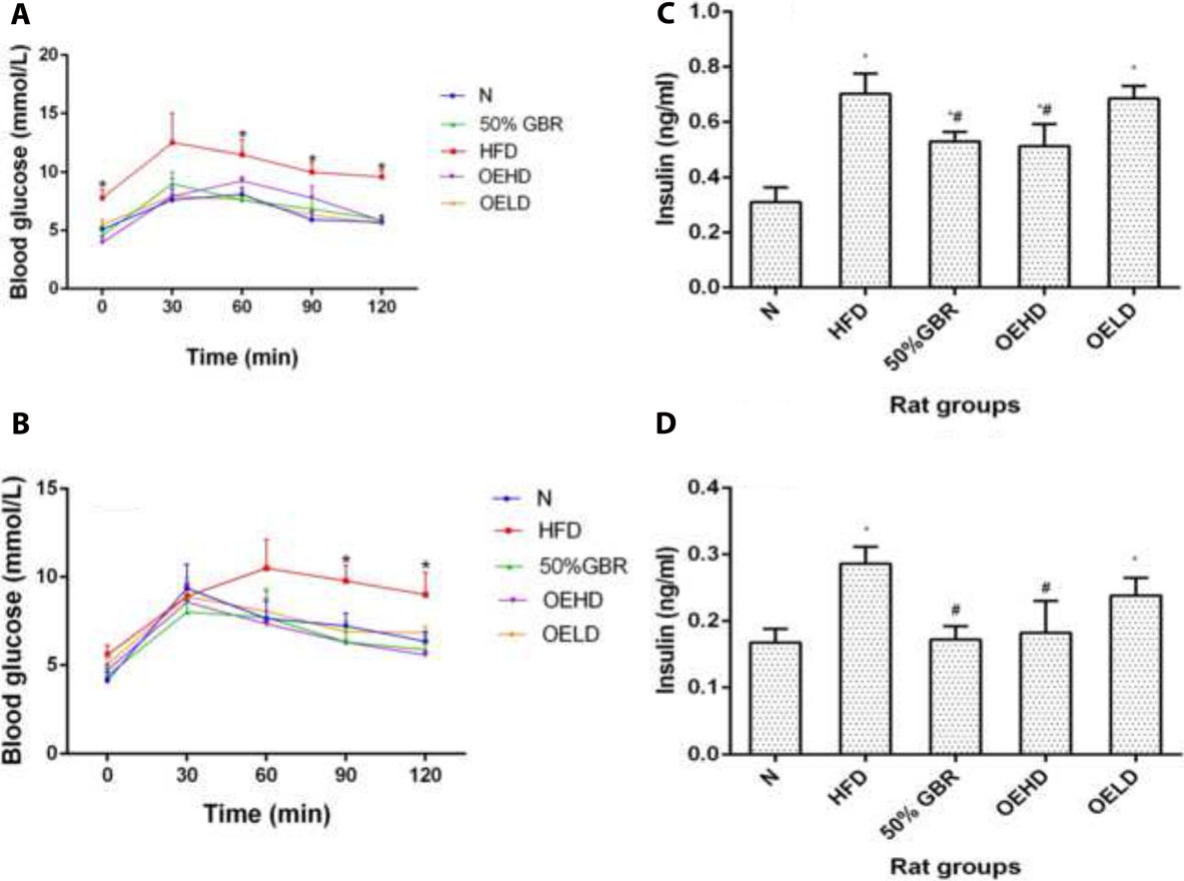
OGTT profiles of **a** dams (mean ± SD, *n* = 3) and **b** offsprings (mean ± SD, *n* = 6), serum insulin levels of dams (**c**) and offsprings (**d**). Data for dams and offsprings represent mean ± SD (*n* = 3) and mean ± SD (*n* = 6), respectively. * Represents significant difference between HFD and treatment groups in dams and offsprings. Bars with ^*^ indicates statistical difference in comparison with HFD group (*p* < 0.05), ^#^ indicates statistical difference in comparison with N group (*p* < 0.05). Groupings are the same as Fig. 1

Blood glucose levels in HFD dams (Fig. 2a) after oral administration of glucose solution (2 g/kg body weight) were highest at 30 min. A significant difference between HFD and the treatment groups was observed at 0 (*P* = 0.00001, *F* = 25.99), 60 (*P* = 0.0002, *F* = 16.54), 90 (*P* = 0.001, *F* = 11.4) and 120 min (*P* = 0.00001, *F* = 54.46) respectively. In the offsprings (Fig. 2b), all the groups peaked at 30 min after oral administration except the HFD group, which peaked at 60 min (*P* = 0.001, *F* = 6.44) and was significantly higher than the rest of the groups at 90 (*P* = 0.00001, *F* = 71.3) and 120 min (*P* = 0.00001, *F* = 38.23).

### Serum insulin

The HFD group had the highest fasting serum insulin level among the dams (Fig. 2c). The GBR and OEHD groups had significantly different levels compared to HFD (*P* = 0.02, *F* = 7.79) and N (*P* = 0.01, *F* = 8.65) groups. In the offsprings (Fig. 2d) fasting serum insulin levels in all the treatment groups differed significantly (*P* = 0.0001, *F* = 36.69) compared to HFD group except OELD group. HFD group had the highest serum insulin, as was the case in the dams.

### Adiponectin

**Fig. 3:**
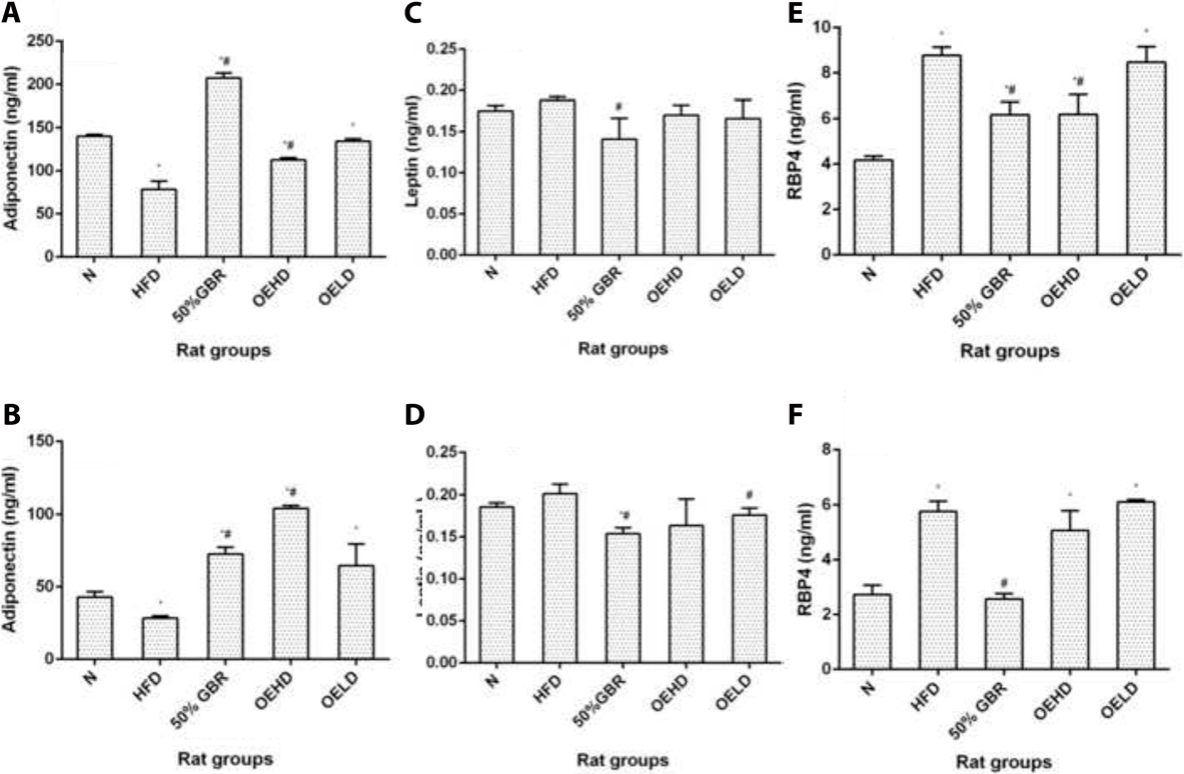
Serum adiponectin levels in dams (**a**) and offsprings (**b**), and serum leptin levels in dams (**c**) and offsprings (**d**), serum retinol binding protein 4 (RBP4) levels of dams (**e**) and offsprings (**f**). Data for dams and offsprings represent mean ± SD (*n* = 3) and mean ± SD (*n* = 6), respectively. Bars with ^*^ indicates statistical difference in comparison with HFD group (*p* < 0.05), ^#^ indicates statistical difference in comparison with N group (*p* < 0.05). Groupings are the same as Fig. 1

In the dams (Fig. 3a), serum adiponectin levels were highest in the GBR group, while the HFD group had the lowest (*P* = 0.00001, *F* = 216.18). The level was also lowest in the HFD offsprings in comparison with other groups, while the OEHD group had the highest level (*P* = 0.00001, *F* = 49) (Fig. 3b).

### Leptin

Leptin level in serum of dams (Fig. 3c) was lowest in the GBR group and highest in the HFD group (*P* = 0.017, *F* = 5.12). A similar trend was observed for the offsprings (*P* = 0.016, *F* = 5.15) (Fig. 3d).

### Retinol binding protein 4 (RBP4)

Serum RBP4 levels in dams (Fig. 3e) were high in the HFD and OELD groups, while they were lower in the N, GBR and OEHD groups compared with the former groups (*P* = 0.00001, *F* = 31.97). A similar pattern was observed in the offsprings (*P* = 0.00001, *F* = 178.75) (Fig. 3f).

### 8 – Iso prostaglandin

**Fig. 4:**
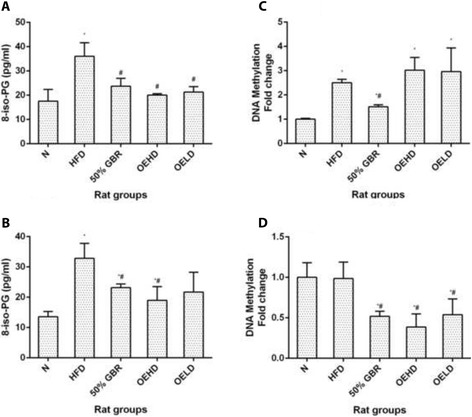
Serum 8-isoprostaglandin (8-iso-PG) levels of dams (**a**) and offsprings (**b**), and fold changes in hepatic global DNA methylation in dams (**c**) and offsprings (**d**). Data for dams and offsprings represent mean ± SD (*n* = 3) and mean ± SD (*n* = 6), respectively. Bars with ^*^ indicates statistical difference in comparison with HFD group (*p* < 0.05), ^#^ indicates statistical difference in comparison with N group (*p* < 0.05). Groupings are the same as Fig. 1

The levels of 8 – Iso prostaglandin in the N and treatment groups in the dams (*P* = 0.0008, *F* = 11.83) (Fig. 4a) and offsprings (*P* = 0.006, *F* = 7.07) (Fig. 4b) were significantly lower than in the HFD group.

### Methylated DNA quantification

The degree of DNA methylation was expressed in fold change with respect to the N group in both dams and offsprings. In the dams (Fig. 4c), the GBR group had significantly lower levels of DNA methylation compared to the HFD group. The OEHD and OELD groups had higher but statistically non-significant levels compared with the HFD group (*P* = 0.0017, *F* = 9.82). In the offsprings (Fig. 4d), however, the GBR, OEHD and OELD groups had lower DNA methylation levels compared with the HFD group (*P* = 0.0006, *F* = 13.05).

### Total histone H3 acetylation

**Fig. 5:**
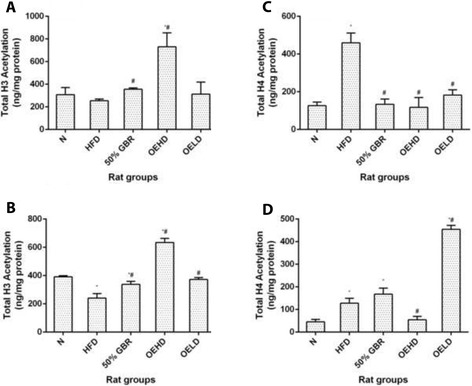
Hepatic total H3 histone acetylation in dams (**a**) and offsprings (**b**), and hepatic total H4 histone acetylation in dams (**c**) and offsprings (**d**). Data for dams and offsprings represent mean ± SD (*n* = 3) and mean ± SD (*n* = 6), respectively. Bars with ^*^ indicates statistical difference in comparison with HFD group (*p* < 0.05), ^#^ indicates statistical difference in comparison with N group (*p* < 0.05). Groupings are the same as Fig. 1

In the dams (Fig. 5a), total histone H3 acetylation (ng/mg protein) levels in the OEHD group were significantly higher than in the HFD group (*P* = 0.00001, *F* = 23.56). The same pattern was observed for the offsprings (*P* = 0.00001, *F* = 134.45) (Fig. 5b).

### Total histone H4 acetylation

All the treatment groups in dams (Fig. 5c) had lower degree of H4 acetylation compared to the HFD group (*P* = 0.00001, *F* = 44.13) while in the offsprings (Fig. 5d), only the N and OEHD groups had lower H4 acetylation compared to the HFD group (*P* = 0.00001, *F* = 268.27).

## Discussion

The occurrence of type 2 diabetes amongst the pediatric population is assuming pandemic proportions, and has been linked with the growing burden of obesity, which disturbs the equilibrium between insulin secretion and sensitivity leading up to insulin resistance [15]. The cause of this pandemic is multifactorial, with several studies indicating that developmental and early life environmental conditions are important variables [16]. Insulin resistance is linked to an increase in lipid accumulation around the liver and in the visceral chambers, often as a result of increased weight gain, and has been shown to correlate with lower levels of adiponectin [17]. In the present study, the GBR groups (dams and offsprings) had low insulin levels with correspondingly high adiponectin levels. Thus, despite the low birth weight observed in GBR and OE groups the offsprings were not at risk of T2D as suggested by the raised adiponectin levels, in favour of improved insulin sensitivity. Additionally, elevated adiponectin levels can promote insulin sensitivity via increasing fat oxidation leading to a reduction of intracellular triglyceride and fatty acid levels in muscle and liver, and overall body weight, similar to what we observed in the present study [18]. As such the enhancements in metabolic indices observed in this study could have been due to elevated adiponectin, in addition to the lower body weights, in both dams and offsprings that consumed GBR and GBR-derived oryzanol. The lower body weights in the offsprings in this study may not have resulted from adverse intrauterine conditions, hence the correspondingly enhanced metabolic conditions. Moreover, we have reported severally that GBR could regulate weight gain and promote weight loss [10], which in the present study may have contributed towards enhanced metabolic outcomes.

As can be recalled, GBR and oryzanol produced metabolic outcomes that favoured insulin sensitivity better than HFD feeding in the dams and offsprings. The dietary fiber present in GBR can lower glycemic index via regulating intestinal glucose absorption, while other bioactives also contribute to better glycemic control through different mechanisms [10, 19]. Oryzanol was also observed to improve glycemic control in mice fed with HFD [20]. Moreover, the metabolic outcomes due to GBR and oryzanol could also have been secondary to lowered leptin levels [21]. Furthermore, there is an inverse correlation in type 2 diabetes between serum adiponectin and 8 – Iso prostaglandin, an important marker of oxidative stress [22], in line with the results we obtained for the HFD dams and offsprings whereby adiponectin levels were low and those of 8 – Iso prostaglandin were raised. Our results ware further supported by the RBP4 levels in the treatment groups, which showed patterns consistent with improved metabolic outcomes [23]. Overall, GBR and oryzanol were able to attenuate the HFD-induced metabolic perturbations in the dams and offsprings, although GBR showed better results suggesting that the presence of multiple compounds conferred superior bioactivity.

In recent years, epigenetic regulation of gene expression has surfaced as a key contributor in the development of metabolic disorders such that unfavorable conditions during the developmental phase (in-utero or lactation) could change the developmental programming of the offspring. In line with this, fetal nutrition is now acknowledged as a crucial regulator of fetal well-being affecting developmental programming [2, 4]. The most studied epigenetic mechanisms include DNA methylation and histone modification with the former involving the methylation at 5’ position of cytosine inside a CpG dinucleotide located at the promoter regions of genes, referred to as CpG islands. Transcriptional repression and activation in these islands is linked to hyper- and hypo-methylation, respectively [4, 24]. Our results showed that a diet of GBR in dams attenuated HFD-induced hepatic global DNA methylation changes and this in turn epigenetically influenced the lower global DNA methylation observed in their offsprings in comparison with the HFD offsprings. Post translational modifications such as acetylation of H3 and H4 also have profound effects on functional activation or repression of genes [24]. Our results also showed that GBR and GBR-derived oryzanol extract modulated H3 and H4 acetylation patterns in dams and their offsprings.

We observed in general a trend in GBR dams, which suggested epigenetically-mediated effects on the offsprings. This trend was less apparent for oryzanol treatment. From our result, it seems plausible that the synergistic effects of the multiple bioactives in GBR on the dams targeting different metabolic pathways were responsible for the attenuation of the HFD-induced metabolic effects in the offsprings, independent of any influences on the EE-BW of the offsprings’ diet. This was also the possible reason why the offsprings’ of the GBR group had better outcomes compared to those of the oryzanol group, although solvent remnants may have contributed some adverse effects to the oryzanol treatment. Similarly, the influence of GBR and oryzanol on gut microbiota of the rats during pregnancy may have contributed to the differential outcomes [2]. Thus, consumption of GBR and oryzanol can be a way to modulate the risk of insulin resistance during pregnancy, the effects of which can be observed in the F1 generation.

## Conclusion

In aggregate, we demonstrated the effects of GBR and oryzanol on HFD-induced insulin resistance and their epigenetic implications in F1 generation of rats. The anti-diabetic effects of GBR and oryzanol have been demonstrated previously, but this is the first report on the epigenetic implications of GBR and oryzanol supplementation in pregnant rats. Our results suggest that GBR and oryzanol can be important functional ingredients for the amelioration of HFD-induced epigenetically-mediated insulin resistance, which may have profound implications on the burden of insulin resistance in humans. These results are worth studying further in the quest for solutions for the raging obesity pandemic.

## Abbreviations

BSA: Bovine serum albumin
GBR: Germinated brown rice
HFD: High fat diet
N: Normal, fed regular chow
OE: GBR-derived gamma oryzanol-rich extract
OEHD: GBR-derived gamma oryzanol-rich extract high dose
OELD: GBR-derived gamma oryzanol-rich extract low dose
OGTT: Oral glucose tolerance test
RBP4: Retinol binding protein
RFU: Relative fluorescence units
